# Therapeutic efficacy of azithromycin, clarithromycin, minocycline and tosufloxacin against macrolide-resistant and macrolide-sensitive *Mycoplasma pneumoniae* pneumonia in pediatric patients

**DOI:** 10.1371/journal.pone.0173635

**Published:** 2017-03-13

**Authors:** Nobuhisa Ishiguro, Naoko Koseki, Miki Kaiho, Tadashi Ariga, Hideaki Kikuta, Takehiro Togashi, Koji Oba, Keisuke Morita, Naoko Nagano, Masanori Nakanishi, Kazuya Hara, Kyosuke Hazama, Toru Watanabe, Tatsuru Yamanaka, Satoshi Sasaki, Hideto Furuyama, Mutsuo Shibata, Satoru Shida, Akihito Ishizaka, Yuichi Tabata, Hayato Aoyagi, Hiroyuki Naito, Mikio Yoshioka, Atsuko Horino, Tsuyoshi Kenri, Hokkaido Pediatric Respiratory Infection Study Group

**Affiliations:** 1 Department of Pediatrics, Hokkaido University Graduate School of Medicine, Sapporo, Hokkaido, Japan; 2 Pediatric Clinic, Touei Hospital, Sapporo, Hokkaido, Japan; 3 Hokkaido Anti–Tuberculosis Association Sapporo Fukujuji Clinic, Sapporo, Hokkaido, Japan; 4 Department of Biostatistics, School of Public Health, Graduate School of Medicine, The University of Tokyo, Tokyo, Japan; 5 Interfaculty Initiative in Information Studies, The University of Tokyo, Tokyo, Japan; 6 Department of Pediatrics, Asahikawa Red Cross Hospital, Asahikawa, Hokkaido, Japan; 7 Nagano Pediatric Clinic, Asahikawa, Hokkaido, Japan; 8 Deparment of Pediatrics, Kushiro Red Cross Hospital, Kushiro, Hokkaido, Japan; 9 Hazama Pediatric Clinic, Muroran, Hokkaido, Japan; 10 Watanabe Pediatric Allergy Clinic, Sapporo, Hokkaido, Japan; 11 Yamanaka Tatsuru Pediatric Clinic, Sapporo, Hokkaido, Japan; 12 Department of Pediatrics, Aiiku Hospital, Sapporo, Hokkaido, Japan; 13 Deparment of Pediatrics, Japan Community Healthcare Organization Hokkaido Hospital, Sapporo, Hokkaido, Japan; 14 Department of Pediatrics, Health Sciences University of Hokkaido, Sapporo, Hokkaido, Japan; 15 Deparment of Pediatrics, Ebetsu Municipal Hospital, Ebetsu, Hokkaido, Japan; 16 Sumiyoshi Kodomo Clinic, Chitose, Hokkaido, Japan; 17 Iwamizawa Pediatric and Gynecology Clinic, Iwamizawa, Hokkaido, Japan; 18 Deparment of Pediatrics, Obihiro Kyokai Hospital, Obihiro, Hokkaido, Japan; 19 Deparment of Pediatrics, Chitose City Hospital, Chitose, Hokkaido, Japan; 20 Deparment of Pediatrics, KKR Sapporo Medical Center, Sapporo, Hokkaido, Japan; 21 Department of Bacteriology II, National Institute of Infectious Diseases, Tokyo, Japan; Chang Gung Memorial Hospital, TAIWAN

## Abstract

**Objective:**

To clarify therapeutic effects of azithromycin, clarithromycin, minocycline and tosufloxacin against macrolide-resistant *Mycoplasma pneumoniae* (MRMP) pneumonia and against macrolide-sensitive *Mycoplasma pneumoniae* (MSMP) pneumonia in pediatric patients.

**Methods:**

A prospective, multicenter observational study was conducted from July 2013 to August 2015. The therapeutic effects of azithromycin, clarithromycin, minocycline and tosufloxacin were evaluated in 59 patients with pneumonia caused by MRMP and in 50 patients with pneumonia caused by MSMP. *In vitro* activities of antimicrobial agents against isolates of *Mycoplasma pneumoniae* were also measured.

**Results:**

Mean durations of fever following commencement of treatment in patients infected with MRMP and MSMP were 5.2 and 1.9 days, respectively (log-rank test, *P* < 0.0001). Among patients infected with MRMP, mean durations of fever were 4.6, 5.5, 1.0 and 7.5 days for patients treated with azithromycin, clarithromycin, minocycline and tosufloxacin, respectively (log-rank test, *P* < 0.0001). Among patients infected with MSMP, mean durations of fever were 2.5, 1.7, 0.9 and 4.3 days for patients treated with azithromycin, clarithromycin, minocycline and tosufloxacin, respectively (log-rank test, *P* = 0.0162). The MIC90s of azithromycin and clarithromycin among the 27 isolates of MRMP were 64 and 256 μg/ml, respectively, and those among the 23 isolates of MSMP were <0.000125 and 0.001 μg/ml, respectively. The MIC90s of minocycline and tosufloxacin among the 27 isolates of MRMP were 1.0 and 0.25 μg/ml, respectively, and those among the 23 isolates of MSMP were 1.0 and 0.5 μg/ml, respectively.

**Conclusion:**

Both minocycline and tosufloxacin showed good *in vitro* activities against MRMP. Minocycline, but not tosufloxacin, shortened the duration of fever in pediatric patients infected with MRMP compared to the duration of fever in patients treated with macrolides.

## Introduction

*Mycoplasma pneumoniae* is one of the common causative pathogens of community-acquired respiratory tract infections mainly in children and young adults [1]. Macrolides are generally considered to be the drugs of choice for treatment of children with *M*. *pneumoniae* infection [2]. Since about 2000, macrolide-resistant *M*. *pneumoniae* (MRMP) has been emerging in Asia, Europe, Canada and the USA [3–6]. The rates of MRMP infection range from 3% to 26% in Europe [7, 8], 63% to 97% in China [9–12] and 25% to 93% in Japan [13–18]. Macrolides are less effective against MRMP infection than against macrolide-sensitive *M*. *pneumoniae* (MSMP) [14, 19, 20]. Recently, the incidence of extra-pulmonary complications in patients with MRMP infection was reported to be significantly higher than that in patients with MSMP infection [21].

Minocycline and fluoroquinolones were shown to be more effective than macrolides in adult patients infected with MRMP [22]. Minocycline and tosufloxacin have also been used for treatment of pediatric patients infected with MRMP [14, 23–26]. Tetracyclines including minocycline are incorporated into teeth, cartilage and bone, resulting in discoloration of both primary and permanent dentitions [27]. Therefore, tetracyclines are contraindicated in children aged less than 8 years [27]. Fluoroquinolones including tosufloxacin have a potential risk of inducing cartilage and joint toxicity in children [28]. Although the Japanese guidelines for management of respiratory infectious diseases in children recommend the use of minocycline or tosufloxacin instead of macrolides when MRMP pneumonia is suspected and when there is a lack of defervescence within 48 h after the initiation of macrolide therapy [29], the clinical effects of tosufloxacin in pediatric patients infected with MRMP have been controversial. One of the reasons for this recommendation was the low MIC titers of minocycline and tosufloxacin against MRMP [14].

The purpose of this study was to clarify the therapeutic effects of macrolides (azithromycin and clarithromycin), minocycline and tosufloxacin against MRMP and MSMP infection in pediatric patients as well as the *in vitro* activities of these antibiotics against MRSP and MSMP.

## Materials and methods

### Ethics statement

All of the necessary ethics approval for this study was obtained from the Institutional Review Board of Hokkaido University Hospital for Clinical Research (012–0174). Written or verbal informed consent was provided by each patient. According to the ethical guidelines for clinical studies in Japan, written informed consent is not necessarily required for research not involving intervention but using human biological specimens. When written informed consent is not obtained, however, the physician must obtain oral informed consent and maintain records of methods for providing information and the content of the information. The acquisition of informed consent was confirmed upon arrival of the clinical specimens. The Institutional Review Board of Hokkaido University Hospital for Clinical Research follows this policy.

### Study design

A prospective, multicenter observational study was conducted from July 2013 to August 2015 at 6 pediatric clinics and in the department of pediatrics in 9 hospitals in Asahikawa, Iwamizawa, Ebetsu, Obihiro, Kushiro, Sapporo, Chitose and Muroran cities, Hokkaido, Japan. Patients aged under 18 years of age who were afflicted with pneumonia due to *M*. *pneumoniae* were enrolled in this study. Diagnosis of pneumonia due to *M*. *pneumoniae* was made when all of the following criteria were met: (1) body temperature above 38 degrees Celsius, (2) signs and symptoms of the respiratory system (cough, dyspnea or abnormal breath sounds), (3) abnormal findings on chest X-ray (lobar or segmental consolidation, tiny centrilobular nodules and bronchovascular thickening) and (4) detection of *M*. *pneumoniae* DNA by real-time PCR (described below) or at least a four-fold increase in IgG antibody against *M*. *pneumoniae* from acute phase serum to convalescent phase serum.

The choice of antibiotics was made according to standard-of-care based on decisions by the treating physicians. The attending pediatrician subsequently chose one of the following antibiotics: azithromycin at 10 mg/kg/day for 3 days, clarithromycin at 10–15 mg/kg/day for 3–7 days, minocycline at 2–4 mg/kg/day for 2–4 days and tosufloxacin at 12mg/kg/day for 3–7 days. Minocycline was not chosen for patients aged less than 8 years because of side effects such as tooth discoloration. Laboratory data including results of a serological test for *M*. *pneumoniae*, radiographic findings, selection and dosage of antibiotics, and outpatient/inpatient status were recorded by the pediatricians. The age and sex of each patient and the time of onset (the first time that the patient had a fever of more than 37.5°C) were recorded by the parents of children. The parents were also instructed to take their children’s axillary body temperatures several times and to record the peak daily body temperatures.

### Real-time PCR assay

Nasopharyngeal swab samples were collected from patients and suspended in three ml of BD universal viral transport medium (Becton Dickinson, Sparks, MD, USA). DNA was extracted with a QIAamp DNA mini kit (Qiagen, Venlo, The Netherlands) from one ml of BD universal viral transport medium and was finally resuspended in 50 μl of a buffer. DNA of *M*. *pneumoniae* was identified by real-time PCR with Mp181-F and Mp181-R primer pairs and an Mp181-P probe using one μl of DNA as described elsewhere [30].

### Detection of macrolide-resistant point mutations in domain V of 23S rRNA gene

Mutations associated with resistance to macrolides at sites 2063, 2064, and 2617 in the *M*. *pneumoniae* 23S rRNA gene domain V region were detected by a sequencing method described elsewhere [31]. *M*. *pneumoniae* showing a point mutation in domain V of the 23S rRNA gene was defined as MRMP.

### Isolation of *M*. *pneumoniae* by culture

Modified Hayflick medium was used for the isolation of *M*. *pneumoniae* from patients [32].

### Antibiotic susceptibility

MICs of antibiotics were determined by a broth microdilution method based on the method of the National Committee for Clinical Laboratory Standards [31].

### Sample size and power calculation

For the primary analysis, 32 patients in each antibiotics group were required for a power of 80% at a two-sided alpha of 0.05 to detect a treatment difference of 1 day with a 1.4 standard deviation regarding fever duration. Therefore, the targeted required number of patients with MRMP infection was 128. The number of patients with *M*. *pneumoniae* infection was estimated to be 50% of those enrolled. The final number of patients with *M*. *pneumoniae* infection was expected to be 256. Since this study has an observational nature, we recruited as many patients as possible from the collaborative clinics and hospitals during the period from July 2013 to August 2015.

### Statistical analysis

Demographic data are expressed as means +- SD or proportions. Continuous variables were compared using Student’s t-test. Frequency analysis was performed by the chi-square test. The distributions of fever duration were depicted by the Kaplan–Meier method, and the log-rank test was used for comparisons of fever duration. To adjust for confounding, we set the duration of fever as a dependent variable and set the following factors as clinically relevant independent variables in multivariate Cox’s regression analysis: age, sex, hospital admission during the course, days from onset of fever to administration of antibiotics, antibiotic initially chosen, change in antibiotics during the course and macrolide resistance of *M*. *pneumoniae*. To be understandable intuitively, we inversed the value of the hazard ratio in this text; that is, a value less than 1.0 means that the duration of fever is shorter than that of the reference. A two-sided *P* value of <0.05 was considered statistically significant. All statistical analyses were performed using JMP software version 12.0.1 (SAS Institute, Cary, NC, USA).

## Results

### Patient characteristics and macrolide resistance of *M*. *pneumoniae*

During the two-year enrollment period, recruitment was slower than expected, and a total of 109 patients who were diagnosed with pneumonia due to *M*. *pneumoniae* were enrolled in this study (S1 Table). In 42 of the 92 *M*. *pneumoniae*-positive samples, the presence of A2063G mutation in the 23S rRNA gene, a single-base mutation that is known to confer macrolide resistance to *M*. *pneumoniae*, was detected, but other mutations (A2063C, A2063T, A2064G and C2617G) were not detected. These mutations were not detected in the remaining 50 *M*. *pneumoniae*-positive samples. Although nasopharyngeal swab samples were not available from seventeen patients who had been shown to have pneumonia due to *M*. *pneumoniae* by serological tests in Kushiro City from July 2013 to January 2014, these patients were regarded as being infected with MRMP (see discussion). The 42 patients from whom MRMP was detected and the 17 patients in Kushiro City in whom *M*. *pneumoniae* infection was serologically demonstrated were incorporated into the MRMP group. Fifty patients from whom *M*. *pneumoniae* was detected but single-base mutations conferring macrolide resistance to *M*. *pneumoniae* were not detected were incorporated into the MSMP group.

No significant differences were found between the MRMP and MSMP patients in baseline status items: age, sex, time from onset of fever to administration of antibiotics and the antibiotic initially chosen (Table 1).

**Table 1:** Background characteristics of patients diagnosed with pneumonia due to *Mycoplasma pneumoniae*.

		MRMP patients	MSMP patients	*P* value
No. of patients		59	50	
Mean age (yr) ± SD (range)		9.0 ± 3.2 (3–17)	9.2 ± 3.3 (2–15)	0.7745
No. of males/females		33/26	29/21	0.8280
Mean duration (days) of fever before administration of antibiotics ± SD (range)		3.1± 1.8 (0–6)	3.6 ± 2.1 (0–8)	0.2056
Antibiotics initially chosen	AZM	18 (30.5%)	11 (22.0%)	0.2217
	CAM	29 (49.2%)	26 (52.0%)	
	MINO	4 (6.8%)	9 (18.0%)	
	TFLX	8 (13.5%)	4 (4.0%)	

AZM, azithromycin; CAM, clarithromycin; MINO, minocycline; TFLX, tosufloxacin; MRMP, macrolide-resistant *Mycoplasma pneumoniae*; MSMP, macrolide-sensitive *Mycoplasma pneumoniae*.

### Antibiotic susceptibility

*In vitro* anti-mycoplasma activities of eight agents against 50 isolates of *M*. *pneumoniae* with or without A2063G mutation in the 23S rRNA gene were measured (Table 2). The MIC90s of erythromycin, clarithromycin, azithromycin and clindamycin among the 27 isolates of MRMP were >256, 256, 64 and 128 μg/ml, respectively, and those among the 23 isolates of MSMP were 0.0039, 0.001, <0.000125 and 0.5 μg/ml, respectively. The MIC90s of levofloxacin, ciprofloxacin, tosufloxacin and minocycline among the 27 isolates of MRMP were 0.5, 1.0, 0.25 and 1.0 μg/ml, respectively, and those among the 23 isolates of MSMP were 0.5, 1.0, 0.5 and 1.0 μg/ml, respectively. The results for resistant gene mutation of the 50 cultural isolates were consistent with those for the original isolates.

**Table 2:** *In vitro* anti-mycoplasma activities against clinical isolates of *M*. *pneumoniae* with or without A2063G mutation in the 23S rRNA gene.

Antimicrobial agent	MIC (μg/ml) for MRMP (n = 27)			MIC (μg/ml) for MSMP (n = 23)		
	Range	50%	90%	Range	50%	90%
Erythromycin	128 - >256	256	>256	0.002–0.0078	0.0039	0.0039
Clarithromycin	64 - >256	256	256	0.0005–0.0039	0.001	0.001
Azithromycin	16–128	32	64	<0.000125–0.00025	<0.000125	<0.000125
Clindamycin	16–256	64	128	0.13–0.5	0.25	0.5
Levofloxacin	0.25–0.5	0.5	0.5	0.25–0.5	0.5	0.5
Ciprofloxacin	0.5–1	1	1	0.5–2	1	1
Tosufloxacin	0.13–0.25	0.25	0.25	0.13–0.5	0.25	0.5
Minocycline	0.13–1	0.5	1	0.13–2	0.5	1

MRMP, macrolide-resistant *Mycoplasma pneumoniae*; MSMP, macrolide-sensitive *Mycoplasma pneumoniae*.

### Duration of fever following commencement of treatment

**Fig 1:**
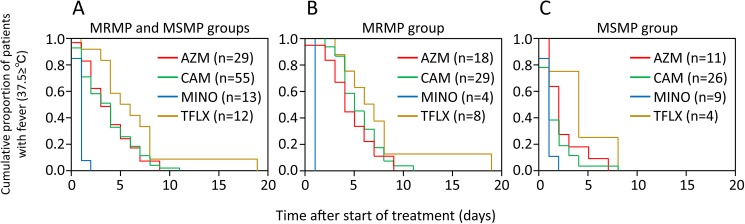
Durations of fever following commencement of treatment for pneumonia due to MRMP and MSMP by azithromycin, clarithromycin, minocycline and tosufloxacin. Kaplan–Meier curves showing a comparison of times taken for body temperature to return to <37.5°C among patients with (A) MRMP and MSMP (log-rank test, *P* < 0.0001), (B) MRMP (log-rank test, *P* < 0.0001) and (C) MSMP (log-rank test, *P* = 0.0162).

The durations of fever following commencement of treatment with azithromycin, clarithromycin, minocycline and tosufloxacin were evaluated by Kaplan-Meier estimates (Fig 1). The mean durations were 3.8, 3.7, 0.9 and 16.4 days for the azithromycin, clarithromycin, minocycline and tosufloxacin groups, respectively (log-rank test, *P* < 0.0001) (Fig 1A). Among the patients with MRMP, the mean durations were 4.6, 5.5, 1.0 and 7.5 days for the azithromycin, clarithromycin, minocycline and tosufloxacin groups, respectively (log-rank test, *P* < 0.0001) (Fig 1B). Among the patients with MSMP, the mean durations were 2.5, 1.7, 0.9 and 4.3 days for the azithromycin, clarithromycin, minocycline and tosufloxacin groups, respectively (log-rank test, *P* = 0.0162) (Fig 1C).

**Fig 2:**
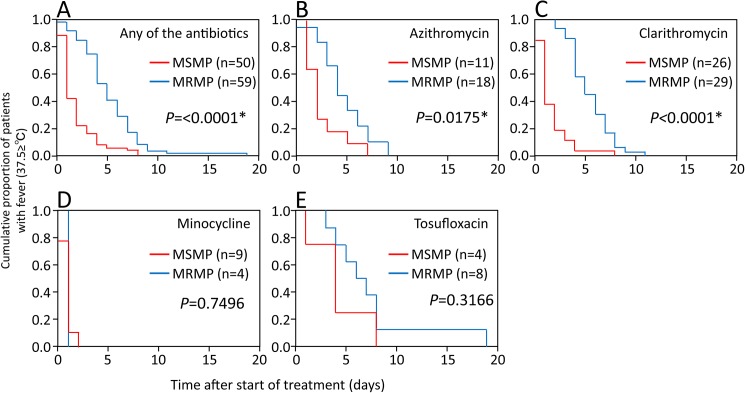
Differences between fever durations in the MRMP and MSMP groups following commencement of treatment with azithromycin, clarithromycin, minocycline and tosufloxacin. Kaplan–Meier curves showing a comparison of times taken for body temperature to return to <37.5°C among patients infected with MRMP and patients infected with MSMP who were treated with (A) any of the antibiotics (log-rank test, *P* < 0.0001), (B) azithromycin (log-rank test, *P* = 0.0175), (C) clarithromycin (log-rank test, *P* < 0.0001), (D) minocycline (log-rank test, *P* = 0.7496) and (E) tosufloxacin (log-rank test, *P* = 0.3166).

The durations of fever following commencement of treatment in patients infected with MRMP and patients infected with MSMP were evaluated by Kaplan-Meier estimates (Fig 2). The mean durations were 5.2 and 1.9 days for the MRMP and MSMP groups, respectively (log-rank test, *P* < 0.0001) (Fig 2A). The durations of fever were significantly different between MRMP patients and MSMP patients treated with azithromycin (4.6 and 2.5 days, respectively, *P* = 0.0175) (Fig 2B) and between MRMP patients and MSMP patients treated with clarithromycin (5.4 and 1.7 days, respectively, *P* < 0.0001) (Fig 2C). No statistically significant difference in the duration of fever was found between MRMP patients and MSMP patients treated with minocycline (1.0 and 0.9 days, respectively, *P* = 0.7496) (Fig 2D) or between MRMP patients and MSMP patients treated with tosufloxacin (7.5 and 4.3 days, respectively, *P* = 0.3166) (Fig 2E).

**Fig 3:**
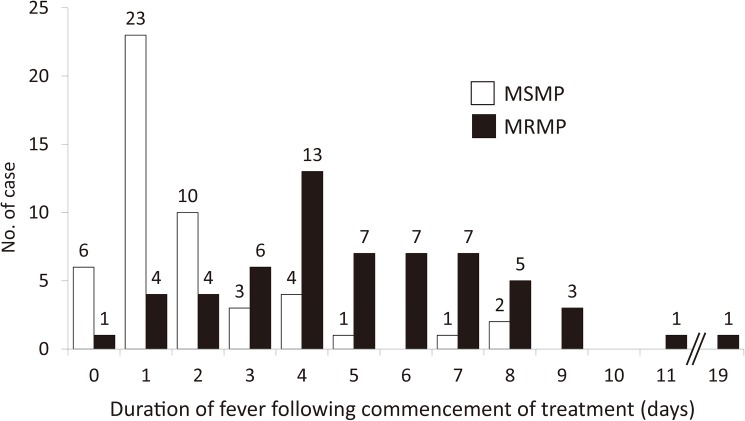
Durations of fever following commencement of treatment for pneumonia due to MRMP and MSMP. Histograms showing the durations of fever following commencement of treatment for pneumonia due to MRMP (black bar) and pneumonia due to MSMP (white bar).

Fever subsided within two days following commencement of treatment in 9 (15%) of the 59 patients infected with MRMP and in 39 (78%) of the 50 patients infected with MSMP (Fig 3).

The Cox proportional hazards model showed that the duration of fever following commencement of treatment correlated with hospital admission during the course (hazard ratio = 0.48, 95% confidence interval of 0.29 to 0.78, *P* = 0.0031) and with macrolide resistance of *M*. *pneumoniae* (hazard ratio = 0.41, 95% confidence interval of 0.26 to 0.64, *P* < 0.0001) (Table 3). There was a statistically significant association between duration of fever following commencement of treatment and the antibiotic initially chosen. Patients who were treated with minocycline had a shorter duration of fever than did patients treated with azithromycin (hazard ratio = 0.35, 95% confidence interval of 0.15 to 0.85, *P* = 0.0215), clarithromycin (hazard ratio = 0.26, 95% confidence interval of 0.11 to 0.61, *P* = 0.0024) or tosufloxacin (hazard ratio = 0.16, 95% confidence interval of 0.06 to 0.43 *P* = 0.0004). Patients treated with azithromycin had a shorter duration of fever than did patients treated with tosufloxacin (hazard ratio = 0.45, 95% confidence interval of 0.20 to 0.91, *P* = 0.0256). There was no statistically significant association between duration of fever following commencement of treatment and age (*P* = 0.9896), sex (*P* = 0.2810), days from fever onset to commencement of treatment (*P* = 0.0621) or change in antibiotics during the course (*P* = 0.2273).

**Table 3:** Results of analysis using the Cox proportional hazards model to determine factors influencing duration of fever following commencement of treatment.

Independent factors	Unit/Control	Patients infected with either with MRMP or MSMP (n = 109)		Patients infected with MRMP (n = 59)		Patients infected with MSMP (n = 50)	
		Hazard ratio (95% Confidence interval)	*P*	Hazard ratio (95% Confidence interval)	*P*	Hazard ratio (95% Confidence interval)	*P*
Age	Per one year	1.00 (0.93–1.07)	0.9896	1.00 (0.91–1.10)	0.9467	1.00 (0.90–1.11)	0.9496
Sex	Female	1.25 (0.83–1.89)	0.2810	1.14 (0.62–2.08)	0.6772	1.69 (0.88–3.25)	0.1075
Admitted patients or outpatients	Admitted patients	0.48 (0.29–0.78)	0.0031	0.50 (0.25–0.98)	0.0404	0.39 (0.17–0.87)	0.0240
Days from fever onset to administration	Per one day	0.89 (0.79–1.01)	0.0621	0.92 (0.76–1.10)	0.3452	0.79 (0.65–0.97)	0.0256
Antibiotic initially chosen							
AZM	CAM	0.73 (0.45–1.22)	0.2279	0.66 (0.35–1.28)	0.2228	0.75 (0.34–1.72)	0.4785
MINO	AZM	0.35 (0.15–0.85)	0.0215	0.03 (0.00–0.20)	0.0003	0.70 (0.21–2.22)	0.5529
MINO	CAM	0.26 (0.11–0.61)	0.0024	0.02 (0.00–0.13)	<0.0001	0.52 (0.18–1.54)	0.2378
AZM	TFLX	0.45 (0.20–0.91)	0.0256	0.48 (0.17–1.27)	0.1410	0.33 (0.08–1.06)	0.0639
CAM	TFLX	0.61 (0.29–1.19)	0.1554	0.72 (0.26–1.82)	0.5060	0.44 (0.12–1.23)	0.1269
MINO	TFLX	0.16 (0.06–0.43)	0.0004	0.01 (0.00–0.11)	<0.0001	0.23 (0.04–1.01)	0.0511
Change in antibiotics during the course	Without change	1.41 (0.82–2.50)	0.2273	1.12 (0.58–2.22)	0.7350	1.72 (0.63–5.56)	0.3269
Macrolide resistance of *M*. *pneumoniae*	Macrolide resistant	0.41 (0.26–0.64)	<0.0001	-	-	-	-

AZM, azithromycin; CAM, clarithromycin; MINO, minocycline; TFLX, tosufloxacin; MRMP, macrolide-resistant *Mycoplasma pneumoniae*; MSMP, macrolide-sensitive *Mycoplasma pneumoniae*.

The durations of fever following commencement of treatment in the 59 patients infected with MRMP were sub-analyzed using the Cox proportional hazards model (Table 3). The duration of fever following commencement of treatment correlated with hospital admission during the course (hazard ratio = 0.50, 95% confidence interval of 0.25 to 0.98, *P* = 0.0404) and with the antibiotic initially chosen. Patients treated with minocycline had a shorter duration of fever than did patients treated with azithromycin (hazard ratio = 0.03, 95% confidence interval of 0.00 to 0.20, *P* = 0.0003), clarithromycin (hazard ratio = 0.02, 95% confidence interval of 0.00 to 0.13, *P* < 0.0001) or tosufloxacin (hazard ratio = 0.01, 95% confidence interval of 0.00 to 0.11, *P* < 0.0001). The durations of fever following commencement of treatment in the 50 patients infected with MSMP were also sub-analyzed using the Cox proportional hazards model (Table 3). The duration of fever following commencement of treatment correlated with hospital admission during the course (hazard ratio = 0.39, 95% confidence interval of 0.17 to 0.87, *P* = 0.0240) and with days from fever onset to administration (hazard ratio = 0.79, 95% confidence interval of 0.65 to 0.97, *P* = 0.0256). There was no statistically significant association between duration of fever following commencement of treatment and age (*P* = 0.9496), sex (*P* = 0.1075), or the antibiotic initially chosen.

## Discussion

In the present study, the therapeutic effects of azithromycin, clarithromycin, minocycline and tosufloxacin against MRMP and MSMP infection in pediatric patients were investigated. In patients treated with macrolides (azithromycin and clarithromycin), the duration of fever following commencement of treatment for MRMP was longer than that following commencement of treatment for MSMP (Figs 2 and 3). These results were consistent with the results of the antibiotic susceptibility tests (Table 2). Similar findings have been reported [14, 19, 20, 24].

In patients infected with MRMP, the duration of fever following commencement of treatment with minocycline was significantly shorter than the duration of fever following commencement of treatment with macrolides (azithromycin and clarithromycin) and tosufloxacin (Fig 1, Table 3). Minocycline is well known to be effective for treatment of MRMP infection in pediatric patients aged more than 8 years [14, 23, 26]. The clinical effects of tosufloxacin in patients infected with MRMP have been controversial. Kawai *et al*. reported that the 48-h defervescence rate after initiation of treatment with tosufloxacin (43 of 62 patients, 69%) was significantly higher than that after initiation of treatment with azithromycin (11 of 27 patients, 41%) [14]. Okada *et al*. reported that 24-h defervescence rates after initiation of treatment with tosufloxacin and macrolides were 31% (4 of 13 patients) and 31% (4 of 13 patients), respectively [26]. In our study, the duration of fever following commencement of treatment with tosufloxacin in patients infected with MRMP was not significantly different from the duration of fever following commencement of treatment with macrolides (azithromycin and clarithromycin) (Fig 1, Table 3). Additionally, the duration of fever following commencement of treatment with tosufloxacin in patients infected with MRMP was almost same as that for patients infected with MSMP (Fig 2). In this study, we could not demonstrate a therapeutic advantage of tosufloxacin for treatment of MRMP.

Both minocycline (MIC90, 1.0 μg/ml) and tosufloxacin (MIC90, 0.25 μg/ml) had higher antibacterial efficacies than those of azithromycin (MIC90, 64 μg/ml) and clarithromycin (MIC90, 256 μg/ml) against isolates of MRMP (Table 2). Therefore, antibacterial efficacy alone could not explain the differences in clinical effects of minocycline and tosufloxacin in patients infected with MRMP. Minocycline has a relatively high blood concentration (2.3 μg/ml after oral administration of 4 mg/kg) and a long half-time (10 hours) [33]. Minocycline penetrates efficiently into lung tissues and bronchial mucus; mean tissue or mucus concentration to plasma concentration ratios were 3.78 +/- 1.10 for lung parenchyma, 4.04 +/- 1.31 for bronchial walls and 1.99 +/- 1.80 for intraluminal mucus collected from bronchi after oral administration of 100 mg for 3 days [34]. In contrast, the maximum blood concentration of tosufloxacin is relatively low (1.0 μg/ml after administration of 6 mg/kg) and its half-time is short (3.8 hours) [35]. Tosufloxacin does not penetrate efficiently into lung sputum and bronchial mucus; maximum sputum concentration to serum concentration ratios were 0.34/0.94 in one patient and 0.31/0.51 in another patient after oral administration of 150 mg [36]. These differences can partially explain the differences in clinical effects of these two antibiotics in MRMP infections.

Seventeen patients in Kushiro City for whom *M*. *pneumoniae* infection was serologically demonstrated but nasopharyngeal swab samples were not available were considered to be patients with MRMP for the following reasons. First, the rate of MRMP infection in Kushiro City was 100% in this period (July 2013 to January 2014) [16]. Second, there was no statistically significant difference in fever duration between MRMP patients and the 17 patients who were shown to have pneumonia due to *M*. *pneumoniae* by serological tests.

In patients infected with MSMP, the durations of fever following commencement of treatments with four antibiotics were different (*P* = 0.0162) (Fig 1). The durations of fever following commencement of treatments with azithromycin, clarithromycin and minocycline were shorter than the duration of fever following commencement of treatment with tosufloxacin, though the difference was not statistically significant (Table 3).

Our study has several limitations. First, this study was a nonrandomized trial to compare the efficacies of several antibiotics against MRMP strains. Selection of antibiotics was made by the attending physicians. The lack of evaluation of pneumonia severity could be a confounding factor for comparison of fever duration and choice of the antibiotic for treating MRMP or MSMP. We started surveillance of MRMP among pediatric patients in Hokkaido in 2012 [16], and physicians therefore knew the prevalence of macrolide resistance in their region. That might have influenced their choice of antibiotic for treating *M*. *pneumoniae* pneumonia. Physicians in Kushiro City were inclined to select either minocycline or tosufloxacin because the macrolide resistance rate in Kushiro City was 100% in that period (July 2013 to January 2014). Six of thirteen patients prescribed minocycline and six of twelve patients prescribed tosufloxacin resided in Kushiro City. Second, we assessed the clinical outcome only by using fever, not by using respiratory symptoms. Third, we could not totally exclude colonization of *M*. *pneumoniae* instead of infection in the *M*. *pneumoniae* DNA-positive patients because of the lack of an IgG antibody against *M*. *pneumoniae* between acute and convalescent phase serum. Finally, we could not achieve the target sample size in this study. If we had calculated the sample size to detect the difference between 3.8 days vs. 0.9 days with 1.4 standard deviation, the required sample size becomes 7 patients with *M*. *pneumoniae* for each antibiotic (28 in total) with 0.5 of 2-sided alpha and 90% power. Thus, this post-hoc power calculation shows that relatively modest differences among groups could be detected with 59 MRMP patients in the present study despite the failure to reach the planned sample size.

In conclusion, minocycline was clinically more effective against MRMP infection in pediatric patients than were the other three antibiotics (azithromycin, clarithromycin and tosufloxacin). Therefore, minocycline could be one of the choices for treatment of MRMP infection in children over 8 years of age. Treatment of MRMP infection in children less than 8 years of age should be investigated.

## Supporting information

S1 Table Data set for this manuscript. (XLS)

## References

[1] Principi N, Esposito S. Macrolide-resistant Mycoplasma pneumoniae: its role in respiratory infection. The Journal of antimicrobial chemotherapy. 2013;68(3):506–11. 10.1093/jac/dks457 23169891

[2] Waites KB, Talkington DF. Mycoplasma pneumoniae and its role as a human pathogen. Clinical microbiology reviews. 2004;17(4):697–728, table of contents. PubMed Central PMCID: PMC523564. 10.1128/CMR.17.4.697-728.2004 15489344 PMC523564

[3] Okazaki N, Narita M, Yamada S, Izumikawa K, Umetsu M, Kenri T, et al Characteristics of macrolide-resistant Mycoplasma pneumoniae strains isolated from patients and induced with erythromycin in vitro. Microbiology and immunology. 2001;45(8):617–20. Epub 2001/10/11. 11592636 10.1111/j.1348-0421.2001.tb01293.x

[4] Li X, Atkinson TP, Hagood J, Makris C, Duffy LB, Waites KB. Emerging macrolide resistance in Mycoplasma pneumoniae in children: detection and characterization of resistant isolates. The Pediatric infectious disease journal. 2009;28(8):693–6. Epub 2009/07/28. 10.1097/INF.0b013e31819e3f7a 19633515

[5] Pereyre S, Charron A, Renaudin H, Bebear C, Bebear CM. First report of macrolide-resistant strains and description of a novel nucleotide sequence variation in the P1 adhesin gene in Mycoplasma pneumoniae clinical strains isolated in France over 12 years. Journal of clinical microbiology. 2007;45(11):3534–9. Epub 2007/09/21. PubMed Central PMCID: PMC2168523. 10.1128/JCM.01345-07 17881549 PMC2168523

[6] Eshaghi A, Memari N, Tang P, Olsha R, Farrell DJ, Low DE, et al Macrolide-resistant Mycoplasma pneumoniae in humans, Ontario, Canada, 2010–2011. Emerging infectious diseases. 2013;19(9). PubMed Central PMCID: PMC3810904. 10.3201/eid1909.121466 PMC3810904 23968896

[7] Dumke R, von Baum H, Luck PC, Jacobs E. Occurrence of macrolide-resistant Mycoplasma pneumoniae strains in Germany. Clinical microbiology and infection: the official publication of the European Society of Clinical Microbiology and Infectious Diseases. 2010;16(6):613–6. Epub 2009/09/22. 10.1111/j.1469-0691.2009.02968.x 19765022

[8] Chironna M, Sallustio A, Esposito S, Perulli M, Chinellato I, Di Bari C, et al Emergence of macrolide-resistant strains during an outbreak of Mycoplasma pneumoniae infections in children. The Journal of antimicrobial chemotherapy. 2011;66(4):734–7. Epub 2011/03/12. 10.1093/jac/dkr003 21393214

[9] Zhou Z, Li X, Chen X, Luo F, Pan C, Zheng X, et al Macrolide-Resistant Mycoplasma pneumoniae in Adults in Zhejiang, China. Antimicrobial agents and chemotherapy. 2015;59(2):1048–51. 10.1128/AAC.04308-14 25451048 PMC4335877

[10] Ma Z, Zheng Y, Deng J, Ma X, Liu H. Characterization of macrolide resistance of Mycoplasma pneumoniae in children in Shenzhen, China. Pediatric pulmonology. 2014;49(7):695–700. 10.1002/ppul.22851 23861188

[11] Liu X, Jiang Y, Chen X, Li J, Shi D, Xin D. Drug resistance mechanisms of Mycoplasma pneumoniae to macrolide antibiotics. BioMed research international. 2014;2014:320801 PubMed Central PMCID: PMC3925631. 10.1155/2014/320801 24592385 PMC3925631

[12] Zhao F, Liu G, Wu J, Cao B, Tao X, He L, et al Surveillance of macrolide-resistant Mycoplasma pneumoniae in Beijing, China, from 2008 to 2012. Antimicrobial agents and chemotherapy. 2013;57(3):1521–3. PubMed Central PMCID: PMC3591905. 10.1128/AAC.02060-12 23263003 PMC3591905

[13] Matsuda K, Narita M, Sera N, Maeda E, Yoshitomi H, Ohya H, et al Gene and cytokine profile analysis of macrolide-resistant Mycoplasma pneumoniae infection in Fukuoka, Japan. BMC infectious diseases. 2013;13:591 PubMed Central PMCID: PMC3883477. 10.1186/1471-2334-13-591 24330612 PMC3883477

[14] Kawai Y, Miyashita N, Kubo M, Akaike H, Kato A, Nishizawa Y, et al Therapeutic Efficacy of Macrolides, Minocycline, and Tosufloxacin against Macrolide-Resistant Mycoplasma pneumoniae Pneumonia in Pediatric Patients. Antimicrobial agents and chemotherapy. 2013;57(5):2252–8. Epub 2013/03/06. 10.1128/AAC.00048-13 23459497 PMC3632908

[15] Miyashita N, Kawai Y, Akaike H, Ouchi K, Hayashi T, Kurihara T, et al Macrolide-resistant Mycoplasma pneumoniae in adolescents with community-acquired pneumonia. BMC infectious diseases. 2012;12:126 Epub 2012/06/02. PubMed Central PMCID: PMC3478186. 10.1186/1471-2334-12-126 22650321 PMC3478186

[16] Ishiguro N, Koseki N, Kaiho M, Kikuta H, Togashi T, Oba K, et al Regional Differences in Rates of Macrolide-Resistant Mycoplasma pneumoniae in Hokkaido, Japan. Japanese journal of infectious diseases. 2015. 10.7883/yoken.JJID.2015.054 26166502

[17] Kawai Y, Miyashita N, Kubo M, Akaike H, Kato A, Nishizawa Y, et al Nationwide surveillance of macrolide-resistant Mycoplasma pneumoniae infection in pediatric patients. Antimicrobial agents and chemotherapy. 2013;57(8):4046–9. PubMed Central PMCID: PMC3719750. 10.1128/AAC.00663-13 23716043 PMC3719750

[18] Meyer Sauteur PM, Unger WW, Nadal D, Berger C, Vink C, van Rossum AM. Infection with and Carriage of Mycoplasma pneumoniae in Children. Front Microbiol. 2016;7:329 PubMed Central PMCID: PMCPMC4803743. 10.3389/fmicb.2016.00329 27047456 PMC4803743

[19] Suzuki S, Yamazaki T, Narita M, Okazaki N, Suzuki I, Andoh T, et al Clinical evaluation of macrolide-resistant Mycoplasma pneumoniae. Antimicrobial agents and chemotherapy. 2006;50(2):709–12. Epub 2006/01/27. PubMed Central PMCID: PMC1366908. 10.1128/AAC.50.2.709-712.2006 16436730 PMC1366908

[20] Matsubara K, Morozumi M, Okada T, Matsushima T, Komiyama O, Shoji M, et al A comparative clinical study of macrolide-sensitive and macrolide-resistant Mycoplasma pneumoniae infections in pediatric patients. Journal of infection and chemotherapy: official journal of the Japan Society of Chemotherapy. 2009;15(6):380–3. Epub 2009/12/17. 20012728 10.1007/s10156-009-0715-7

[21] Zhou Y, Zhang Y, Sheng Y, Zhang L, Shen Z, Chen Z. More complications occur in macrolide-resistant than in macrolide-sensitive Mycoplasma pneumoniae pneumonia. Antimicrobial agents and chemotherapy. 2014;58(2):1034–8. PubMed Central PMCID: PMC3910883. 10.1128/AAC.01806-13 24277047 PMC3910883

[22] Miyashita N, Akaike H, Teranishi H, Ouchi K, Okimoto N. Macrolide-resistant Mycoplasma pneumoniae pneumonia in adolescents and adults: clinical findings, drug susceptibility, and therapeutic efficacy. Antimicrobial agents and chemotherapy. 2013;57(10):5181–5. PubMed Central PMCID: PMC3811443. 10.1128/AAC.00737-13 23896480 PMC3811443

[23] Komatsu H, Tsunoda T, Inui A, Sogo T, Fujisawa T. Characteristics of hospitalized children infected with macrolide-resistant Mycoplasma pneumoniae. The Brazilian journal of infectious diseases: an official publication of the Brazilian Society of Infectious Diseases. 2014;18(3):294–9. 10.1016/j.bjid.2013.09.004 PMC9427443 24389284

[24] Kawai Y, Miyashita N, Yamaguchi T, Saitoh A, Kondoh E, Fujimoto H, et al Clinical efficacy of macrolide antibiotics against genetically determined macrolide-resistant Mycoplasma pneumoniae pneumonia in paediatric patients. Respirology. 2012;17(2):354–62. Epub 2011/11/15. 10.1111/j.1440-1843.2011.02102.x 22077195

[25] Sakata H. [Clinical efficacy of tosufloxacin in children with pneumonia due to Mycoplasma pneumoniae]. The Japanese journal of antibiotics. 2012;65(3):173–9. Epub 2012/11/24. 23173293

[26] Okada T, Morozumi M, Tajima T, Hasegawa M, Sakata H, Ohnari S, et al Rapid effectiveness of minocycline or doxycycline against macrolide-resistant Mycoplasma pneumoniae infection in a 2011 outbreak among Japanese children. Clinical infectious diseases: an official publication of the Infectious Diseases Society of America. 2012;55(12):1642–9. Epub 2012/09/14. 22972867 10.1093/cid/cis784

[27] Sanchez AR, Rogers RS 3rd, Sheridan PJ. Tetracycline and other tetracycline-derivative staining of the teeth and oral cavity. Int J Dermatol. 2004;43(10):709–15. 10.1111/j.1365-4632.2004.02108.x 15485524

[28] Grady RW. Systemic quinolone antibiotics in children: a review of the use and safety. Expert Opin Drug Saf. 2005;4(4):623–30. 10.1517/14740338.4.4.623 16011441

[29] Committee for the Guidelines in Management of Respiratory Infectious Diseases in Children. Guidelines for the management of respiratory infectious diseases in children in Japan. Tokyo, Japan: Kyowa Kikaku Ltd.; 2011.(in Japanese)

[30] Winchell JM, Thurman KA, Mitchell SL, Thacker WL, Fields BS. Evaluation of three real-time PCR assays for detection of Mycoplasma pneumoniae in an outbreak investigation. Journal of clinical microbiology. 2008;46(9):3116–8. Epub 2008/07/11. PubMed Central PMCID: PMC2546712. 10.1128/JCM.00440-08 18614663 PMC2546712

[31] Matsuoka M, Narita M, Okazaki N, Ohya H, Yamazaki T, Ouchi K, et al Characterization and molecular analysis of macrolide-resistant Mycoplasma pneumoniae clinical isolates obtained in Japan. Antimicrobial agents and chemotherapy. 2004;48(12):4624–30. Epub 2004/11/25. PubMed Central PMCID: PMC529214. 10.1128/AAC.48.12.4624-4630.2004 15561835 PMC529214

[32] Hayflick L. Tissue cultures and mycoplasmas. Texas reports on biology and medicine. 1965;23:Suppl 1:285+. 5833547

[33] Nakazawa S, Oka S, Sato H, Watanabe O, Niino K. [Effects of dry syrup minocycline (Minomycin 'Lederle') in the pediatric field]. The Japanese journal of antibiotics. 1972;25(5):288–94. 4539520

[34] Naline E, Sanceaume M, Toty L, Bakdach H, Pays M, Advenier C. Penetration of minocycline into lung tissues. Br J Clin Pharmacol. 1991;32(3):402–4. PubMed Central PMCID: PMCPMC1368539. 1777379 10.1111/j.1365-2125.1991.tb03920.x PMC1368539

[35] Sunakawa K, Iwai N, Iwata S, Ouchi K, Sakata H, Suzuki K, et al Population pharmacokinetics and pharmacodynamics of tosufloxacin granules in pediatric infectious diseases. Jpn J Chemother. 2010;58(S-2):69–77.

[36] Nasu M, Yamazaki T, Yamazaki H, Kuroda Y, Goto Y, Shigeno H, et al T-3262 in respiratory tract infections. Chemotherapy. 1988;36(S-9):699–709.(in Japanese)

